# Can long-term dentine bonding created in real life be forecasted by parameters established in the laboratory?

**DOI:** 10.1038/srep37799

**Published:** 2016-11-25

**Authors:** Heleine M. C. Rêgo, Thaís S. Alves, Eduardo Bresciani, Li-na Niu, Franklin R. Tay, César R. Pucci

**Affiliations:** 1 Institute of Science and Technology, University of Estadual Paulista, São Paulo, Brazil; 2 State Key Laboratory of Military Stomatology, The Fourth Military Medical University, Xi’an, Shaanxi, P.R. China; 3 College of Graduate Studies, Augusta University, Augusta, GA, USA

## Abstract

Tooth-coloured plastic dental fillings secured by adhesives to tooth structures are widely used to fix decayed teeth. Whereas laboratory tests demonstrate rapid deterioration of the ability of the adhesives to stick to dentine, clinical studies show that these fillings are relatively durable. This discrepancy suggests that the parameters used for simulating bond degradation in the laboratory setting do not correlate well with clinical outcomes. The present study examined the long-term tensile bond strength of resin composite fillings performed in real life and under different laboratory-simulated bonding conditions to identify parameters that may be used to forecast the durability of adhesive bonds created in dentine. Fillings placed *in vivo* were subjected to different periods of intraoral function. *In vitro* specimens were bonded based on whether simulated pulpal pressure (SPP) or thermomechanical cycling was implemented, and how long the completed fillings were stored in water. Thermomechanical cycling used in combination with long-term water ageing are useful in forecasting the decline in strength of resin-dentine bonds created *in vivo*. These parameters should be adopted for future evaluations. Conversely, the use of SPP does not appear to be a significant parameter in the simulation of long-term clinical deterioration of bond integrity.

Tooth decay is the most prevalent chronic diseases worldwide^1^. Every year, millions of people with decayed teeth seek the service of dentists to have their cavities filled with tooth-coloured plastic fillings. These fillings stick to cavities after removal of the bacteria-infected tissues via adhesives that micromechanically or chemically unite the plastic with the hard tissues of a tooth; the latter consists predominantly of enamel and dentine. Although different types of materials have been used for filling decayed teeth, plastic fillings have become popular over the last half-century because they stick and blend well to teeth without showing unnatural colours during smile. Unlike the placement of metallic fillings, the process of filling tooth cavities with plastic resin composites is more technically-demanding^2^,^3^. Since the pioneering work of Buonocore on adhesive bonding to enamel and Brudevold *et al*. on adhesive bonding to dentine^4^,^5^, there has been a phenomenal increase in the bond capability of adhesive filling materials to tooth substrates, in particular, to dentine. Nevertheless, after six decades of intensive research, there are still many limitations associated with bonding to dentine^6^, which is regarded by clinical scientists as a living component of the human body^7^,^8^. The morphological and physical variations of human dentine bring about difficulties in maintaining durable bonding to that substrate^9^. Biological and clinical factors such as dentine permeability, disease-modified dentine, and pulpal fluid flow in a living tooth adversely affect adhesion to dentine^10^.

Pragmatically speaking, oral conditions are not stable. Tooth fillings are constantly subjected to ageing through temperature changes and stresses created by biting and chewing. Body fluids derived from the dental pulp, the living central part of a tooth, can reach the surface of dentine through tubular structures that host the neurological communications between the tooth and its external environment. In real life, the dentinal fluid acts in concert with water derived from cavity rinsing or from saliva, to expedite degradation of the adhesive joint^11^,^12^,^13^. Although the adhesives used for sticky plastic materials to teeth have improved extensively through eight generations of modifications and exhibit acceptable immediate performance, the longevity of dentine bonded with contemporary adhesive systems is highly variable^14^,^15^,^16^,^17^. Clinical studies reported decrease in bond strength arising from degradation of the adhesive joint, especially for adhesion to dentin, with the consequent need for filling replacement^18^,^19^.

It is not always possible to conduct clinical trials on adhesive plastic tooth fillings due to operator variations, lack of patient cooperation and cost constraints from the manufacturers’ perspective. Although far from being the gold standard, laboratory testing has frequently been utilised to fulfil pre-launching criteria of new dental adhesive products. These *in vitro* tests should faithfully reproduce the oral conditions to provide greater reliability in predicting clinical performance^20^. Whereas laboratory tests frequently demonstrate rapid deterioration of the ability of the adhesives to stick to dentine^6^, clinical evaluations of plastic fillings indicated that the fillings appear to be relatively durable^19^,^21^,^22^. This discrepancy suggests that the parameters currently used for simulating bond degradation in the laboratory setting do not correlate well with clinical outcomes^7^.

Here, the strength of the dentine adhesive joint in class I resin composite plastic fillings bonded was evaluated under *in vivo* and *in vitro* conditions. Because cavities are small and custom-prepared by hand drilling, each cavity differs slightly from the other. Hence a miniature technique for challenging the adhesive joint to failure under tension, known as the microtensile bond strength (MTB) test, was used for testing standardised segments obtained from the filled cavities^23^. Both short-term (24 hours) and long-term (6 months) bond strengths of those fillings to dentine were evaluated. Admittedly, a 6-month period of functioning inside the mouth cannot be regarded as long-term over the life-span of a plastic tooth filling. However, one has to take into account that *in vivo* evaluations involve sacrificing teeth that have been functioning in the mouth, which can only be achieved when those teeth are destined for extraction as part of the volunteers’ ongoing dental treatment programmes. Prior to determining whether *in vitro* bonding conditions can replicate *in vivo* bonding effectiveness after intraoral function, one has to first ascertain that the bond strengths of fillings performed *in vivo* are susceptible to weakening after real life functional challenges. One also has to validate that bond strengths of fillings performed in the laboratory will undergo deterioration after adoption of specific bond ageing parameters. Accordingly, three null hypotheses were tested: 1) the time of intraoral functioning has no adverse effect on the bond strength to dentine in fillings performed *in vivo*; 2) the use of simulated pulpal pressure (SPP), thermomechanical ageing and an extended storage period for *in vitro* bond testing have no adverse effect on the bond strength to dentine; 3) *In vitro* testing conditions are incapable of reproducing the long-term dentine bond strength of plastic fillings performed *in vivo* after functional challenges.

## Results

Means and standard deviations of MTB derived from *vivo* and *in vitro* testing are presented in Table 1. For *in vivo* testing, MTB at 24 h (23.21 ± 3.91 MPa) was significantly higher than MTB at 6 months (14.17 ± 4.78 MPa; p < 0.001). Results of the three-way ANOVA for *in vitro* testing conditions indicated that MTB was significantly reduced in the presence of SPP (p < 0.001), thermomechanical cycling (p < 0.05) and after water storage for 6 months (p < 0.001). The interaction between SPP and storage time was significant (p < 0.001). Post-hoc Tukey tests indicated decrease in MTB (in MPa) for groups bonded with SPP (17.34 ± 3.66 for SPP and 20.94 ± 4.61 for no-SPP), after ageing in water (18.40 ± 4.61 for water ageing and 19.87 ± 3.53 for cyclic ageing), and after 6 months of water storage (16.76 ± 4.06 for 6 months and 21.52 ± 3.65 for 24 h).

The interaction between SPP and storage time was significant (Table 1) and was attributed to the low MTB values observed for groups with SSP at 24 h; MTB values were not significantly different for groups that were tested after 6 months. Comparisons among *in vivo* and *in vitro* conditions at 24 h revealed significant differences among the various groups (p < 0.001). After 6 months of water storage, no significant difference was observed (p > 0.05). Significant difference between *in vivo* and *in vitro* conditions was observed at 24 h; MTB values were significantly lower when SPP was employed for *in vitro* bonding (Table 1).

**Figure 1:**
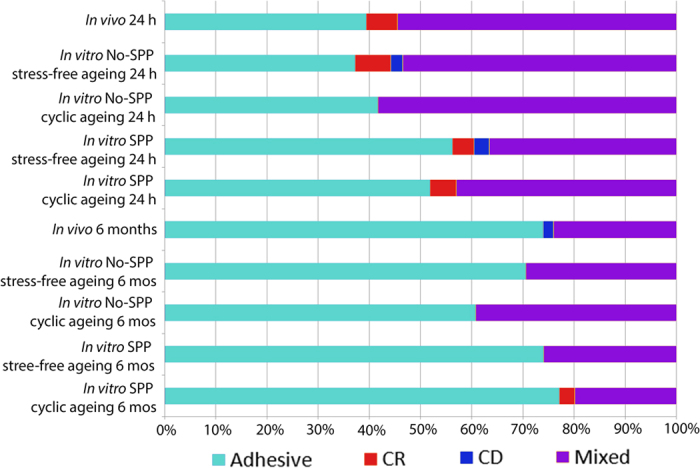
Prevalence (expressed as percentages) of failure types after microtensile bond strength testing for all groups. Adhesive: adhesive failure; CR: cohesive failure in resin; CD: cohesive failure in dentine; Mixed: mixed failure. SPP: simulated pulpal pressure; MOS: months.

**Figure 2:**
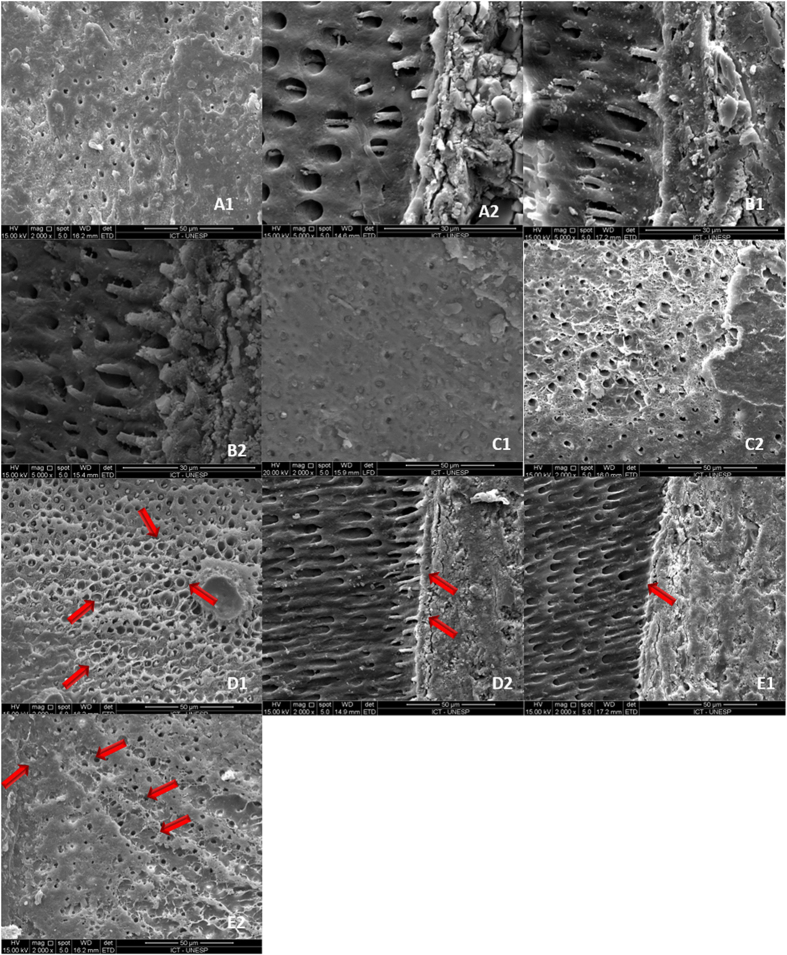
Scanning electron microscopy images for all groups. A1) *in vivo* 24 hours; A2) *in vivo* 6 months; B1) without SPP storage in water - 24 hours; B2) without SPP storage in water - 6 months; C1) without SPP after thermomechanical cycling - 24 hours; C2) Without SPP after thermomechanical cycling - 6 months; D1) With SPP storage in water - 24 hours; D2) With SPP storage in water - 6 months; E1) With SPP after thermomechanical cycling - 24 hours; E2) With SPP after thermomechanical cycling - 6 months. Arrows indicate presence of bubbles on the dentine surface of fractured beams (D1 and E2) and on adhesive interface area of dentine slices (D2 and E1) in the groups in which SPP was performed.

Failure mode analysis showed a larger number of adhesive failure after 6 months of *in vivo* functioning, and for specimens bonded *in vitro* in the presence of SPP (Fig. 1). Scanning electron microscopy (Fig. 2) identified bubbles along the adhesive interface and within the adhesive layer in the SPP groups for both 24 h and 6 month water storage times. No alteration of the adhesive interface was noted for the *in vivo* specimens and *in vitro* specimens bonded in the absence of SPP.

## Discussion

Although bond strength tests are helpful for predicting clinical outcomes, the exact laboratory testing conditions that are useful in reflecting clinical outcome had not been elucidated. To reproduce oral conditions in bench-top bond strength testing, plastic fillings placed in extracted teeth may be stored in different media such as saliva, water, oil or ethanol for different time periods to simulate the effects of different beverage categories. Thermal, mechanical or thermomechanical cycling may be conducted to reproduce changes in materials caused by ageing inside the mouth^24^. Pulpal pressure may be reproduced in the laboratory to simulate dentine moisture there are present in real life^25^. Bond testing may be performed immediately or after long-term ageing to predict the clinical performance of the materials^21^.

In the present study, lower MTB values were recorded for both *in vivo* and *in vitro* bonding conditions, compared to studies on bonding dentine with or without SPP^26^,^27^, although different materials were tested in the present study. Whereas the previous studies tested adhesion to flat dentine surfaces, the present study tested adhesion in filled class I cavities to simulate *in vivo* tooth preparation. This difference in methodology probably accounts for the present lower values. The discrepancy may be explained by the higher cavity configuration factor in class I cavities, that results in the development of higher stresses created on the cavity walls due to polymerisation shrinkage of the plastic filling material^28^,^29^. Incomplete relief these residual stresses, particularly prior to water sorption in the 24 h groups^30^, may have resulted in decreased bond strength.

For the *in vivo* specimens, significant decline in bond strength was observed after 6 months of intraoral function. Hence the first null hypothesis that “the time of intraoral functioning has no adverse effect on the bond strength to dentine in fillings performed *in vivo*” has to be rejected. Decrease in bond strength is probably caused by degradation of resin-sparse collagen fibrils and hydrolysis of resin within the dentine-adhesive joint^31^.

For the *in vitro* specimens, significant decline in MTB values were observed when bonding was performed in the presence of SPP (fluid transudation parameter), after thermomechanical cycling (aging parameter), and after 6 months of water storage (storage time parameter). The interaction between SPP and storage time was also statistically significant; higher MTB was observed at 24 hours in the absence of SPP, whereas SSP did not affect MTB after 6 months. Thus the second null hypothesis that “the use of simulated pulpal pressure (SPP), thermomechanical ageing and an extended storage period for *in vitro* bond testing have no adverse effect on the bond strength to dentine” has to be rejected.

The significantly lower MTB observed in groups bonded in the presence of SPP supports the results of previous studies that examined the effects of dentine permeability and pressure-induced fluid transudation on the strength of the interfacial bond created by etch-and-rinse adhesive systems^32^,^33^,^34^. Despite the importance of water to prevent the collapse of collagen fibrils and to facilitate infiltration of adhesive monomers into demineralised dentine, excessive moisture derived from a vital dental pulp, as reproduced by SPP, might have hindered the interaction between adhesive and dentine^35^. Water-rich zones have been identified within the dentine-adhesive interface that represent entrapment of excess, incompletely removed moisture. These water-rich zones appear as fractal-like patterns or water bubbles within the polymerised adhesive^36^,^37^. These highly water-permeable sites could be identified as voids in the SEM images (Fig. 2), on the dentine surface of the fractured beams and on the adhesive interfacial area of dentine slices. The presence of these interfacial stress raisers increases the ease of crack propagation through the adhesive interface and results in decreased bond strength.

Regarding the choice of *in vitro* ageing protocols, similar MTB was observed for specimens were stored in water only and those there were subjected to thermomechanical cycling. The cyclic ageing process creates stresses along the adhesive-dentine interface via temperature changes and Hertzian contact mechanics to simulate intraoral functioning of the adhesive joint^24^. Other authors also observed decreases in adhesive-dentine bond strengths following thermomechanical cyclic ageing^27^,^38^. Hence, both water ageing and themomechanical cycling were employed in *in vitro* experiments to simulate the clinical conditions because the use of these protocols provided similar results to clinical conditions in the present study.

Regarding storage time (Table 1), bond strength decreased when SPP was utilised during bonding, regardless of storage time. At 24 h, groups bonded under SPP yielded significantly lower bond strength compared with groups that were bonded in the absence of SPP. These results are agreeable with the literature; no difference in bond strength was detected when specimens bonded under SPP were water-aged for 24 h, 6 months or 1 year^34^,^39^. The similarity in MTB among groups bonded under SPP at 24 h, and those groups bonded with or without SPP at 6 months, suggests that degradation caused by SPP is as detrimental as long-term water storage without additional laboratory-derived challenges. Intrinsic moisture derived from the dental pulp prevents adhesive monomers from fully penetrating into acid-etched dentine and inhibits the formation of an optimal adhesive joint^40^. Dilution of water-soluble resin components and phase separation of water-immiscible resin components may also occur in the presence of excessive moisture^41^.

When *in vivo* and *in vitro* bonding results were compared, significant difference was identified between the two bonding scenarios at 24 h but not after 6 months. This leads to rejection of the third null hypothesis that “*In vitro* testing conditions are incapable of reproducing the long-term dentine bond strength of plastic fillings performed *in vivo* after functional challenges”. Among the 24 h groups (Table 1), no difference was observed between the *in vivo* bonding results and the *in vitro* protocols without the use of SPP. This shows that only the *in vitro* bonding protocols without involving SPP were capable of reproducing the 24 h *in vivo* bonding results^24^. Conversely, the 24 h *in vitro* results in the SPP groups were significantly lower than then 24 h *in vivo* bonding result. This suggests that the use of SPP *in vitro* has underestimated short-term *in vivo* bond strength. Simulated pulpal pressure should thus be avoided when assessing immediate (24 hours) bond strength of resin composites to dentin. Longer interval (6 months) assessments may incorporate SPP to simulate the clinical conditions. In the *in vivo* part of the present study, volunteers received local anesthesia containing vasoconstrictor prior to the cavity preparation. Vasoconstrictors can reduce pulpal blood flow to the extent of producing null physiological pulpal pressure^42^. Blood flow reduction prevents fluid transudation through the dentinal tubules to the dentine surface^43^. This may account for the difference in bond strength in *in vitro* specimens bonded in the presence of PPS (physiological pulpal pressure of 1.47 kPa) and those specimens bonded *in vivo* (close to zero physiological pulpal pressure) at 24 h. Short-term differences attributed to the application of *in vitro* SPP, however, were nullified after water storage for 6 months. The results indicate that degradation of resin-dentine bonds by endogenous enzymes present in dentine, and resin hydrolysis caused by water sorption, are far more detrimental to the integrity of dentine-adhesive joints created *in vitro* and *in vivo*, than the use of SSP to simulate the effects of water movement during dentine bonding in the laboratory setting.

The significance of the present work resides on the delineation of clinically-relevant *in vitro* bond testing parameters to facilitate evaluation of the durability of dentine bonding performed in real life. Thermomechanical cycling used in combination with long-term water ageing are useful in forecasting the durability of resin-dentine bonds created *in vivo*. These parameters should be adopted in future evaluations. Conversely, the use of SPP during bench-top bonding procedures does not appear to be a significant parameter in the simulation of long-term clinical deterioration of bond integrity.

## Materials and Methods

The present study was approved by the local Institutional Review Board of the University of Estadual Paulista, Brazil (protocol 79,921). The methods were carried out in accordance with the principles of the Declaration of Helsinki on experiments involving human subjects. Informed written consent of the human volunteers participating in the study was obtained. Forty class I resin restorations in third molars were performed *in vivo* and remained in service for 24 h or 6 months prior to MTB evaluation.

For *in vitro* testing, eighty extracted human posterior teeth were divided into two fluid transudation groups (n = 40): SPP (restoration performed under SPP) and no-SPP (restoration done without SPP). Each of the two groups were divided two ageing subgroups (n = 20): water ageing (water storage only); or cyclic ageing (thermomechanical cycling prior to water storage). Each of the subgroups was further subdivided into two storage time subdivisions (n = 10): storage in water for 24 h or 6 months. The materials employed for filling the cavities are listed in Table 2.

### *In vivo* protocol

#### Patient selection

Inclusion criteria consisted of both male and female volunteers with age above 18 years, presenting with good oral hygiene, low caries index, absence of periodontal diseases, parafunctional habits, or partial dentures. The selected posterior teeth were planned for extraction based on orthodontic treatment decisions. Those teeth were not presented with clinical or radiographic signs and symptoms of irreversible pulpal changes. In addition, the antagonist and neighbouring teeth were present.

#### Cavity preparation and filling

Tooth cleaning was first performed for the quadrant containing the selected tooth. Local anesthesia was administered with 2% mepivacaine with vasoconstrictor. Cavity preparation was performed by a single operator with a cylindrical diamond bur inserted in a high-speed dental drill with constant water cooling. The dimension of the cavity preparation was 4 mm in length, 5 mm wide and 2 mm deep considering the marginal ridge as reference.

During the tooth filling procedures, each tooth was prevented from saliva contamination using a dam made of rubber. The cavity preparation was conditioned with 37% phosphoric acid for 30 sec on enamel and 15 sec on dentine. The acid-etched cavity was rinsed with water for 30 sec and dentine moisture was controlled with cotton pellets. Single Bond 2 adhesive was applied according to the manufacturer’s instructions. Each bonded cavity was filled with a resin composite plastic material. The plastic filling material was inserted into the cavity using an incremental technique (2 mm/increment). Each increment was light-cured for 40 sec using a light equipped with light-emitting diode (440–480 nm wavelength range) with a power output of 1200 mW/cm^2^ (RADII-CAL, SDI, Melbourne, Victoria, Australia). After finishing and polishing, the teeth remained in service for 24 h or 6 months prior to extraction. Each tooth was cleaned and stabilised with acrylic resin prior to MTB testing.

#### *In vitro* protocol

Non-carious human extracted third molars due to orthodontic or periodontal reasons were used. Cavity preparation and tooth filling procedures were the same as the *in vivo* group. Each tooth was similarly stabilised with acrylic resin with the crown of the tooth free of acrylic resin.

#### Simulated Pulpal Pressure

Pulpal pressure was simulated by using a device adapted from the University of Zurich (Switzerland). The device contains a water reservoir positioned 20 cm above the pulp chamber of the tooth to simulate physiological pulpal pressure of 1.47 kPa^32^. Inflow and outflow of water from the pulp chamber was performed using two hypodermic needles connected via two silicone cannulae (Rush, Dublin, Ireland).

#### Ageing

The filled teeth in the water-ageing subgroup were stored in distilled water at 37 °C for 24 h or 6 months. Water was changed once a week during the 6 month period. The filled teeth in the cyclic aging subgroup were first stored for 24 h in distilled water to ensure complete curing of the resin composite. The specimens were stressed with 27 thermal cycles (10 °C for 1 min, 25 °C for 1 min and 55 °C for 1 min) and 666 mechanical cycles, corresponding to 24 h of intraoral function; or 5,000 thermal cycles and 120,000 mechanical cycles, corresponding to 6 months of intraoral function^44^,^45^. Themocycling was performed in a thermomechanical wear system (model ER-37000; Erios, São Paulo, SP, Brazil).

### MTB testing

After treatment, specimen was sectioned parallel to the longitudinal axis of the tooth. Sectioning was performed in the buccolingual direction into slices, and then in the mesiodistal direction in beams, using a precision cutting machine with water cooling (Labcut 1010; Extec, Enfield, CT, USA). This generated an average of 9 beams per tooth, each containing the adhesive joint between the plastic filling and dentine. The dimension of each beam was approximately 1 mm × 1 mm × 8 mm; the exact length and breadth of the adhesive joint was precisely measured for bond strength derivatisation. One slice per group was used for scanning electron microscopy. Microtensile testing was performed in a universal testing machine using a 10 kg load cell at a crosshead speed of 0.5 mm/min. After bond testing, the fractured beams were evaluated using a stereomicroscope at 50x magnification to record the failure mode.

### Scanning Electron Microscopy (SEM)

The dentine portion of representative fractured beams with adhesive fracture, and dentine slices obtained from initial sectioning of the teeth were used for SEM (JMS 5310; JEOL, Tokyo, Japan). Specimens were dehydrated, sputter-coated with gold/palladium, and examined at 15 kV. The images were used to clarify the classification of type of failure, for selection of the data to be included in the statistical analysis (see below).

### Statistics Analyses

Data generated from beams with cohesive failure in the plastic filling material or tooth structure were discarded and only data from beams with adhesive or mixed failure were analysed. The mean MTB of beams derived from one tooth was used to represent the tensile bond strength of that tooth, yielding ten values per group/subgroup/subdivision.

Data were analysed with parametric statistical methods after validating the normality and homoscedasticity of the data sets. If those assumptions were violated, nonlinear transformations were performed to satisfy the assumptions prior to the use of parametric testing methods. One-way analysis of variance (ANOVA) was employed for analysing “storage time” for *in vivo* testing. Three-way ANOVA was employed for analysing the parameters involved in the *in vitro* testing (SPP, ageing protocol and storage time). One-way ANOVA was employed to compare *in vivo* and *in vitro* conditions. Post-hoc pairwise comparisons were performed using the Tukey test. For all analyses, statistical significance was set at α = 0.05.

## Additional Information

**How to cite this article**: Rêgo, H. M. C. *et al*. Can long-term dentine bonding created in real life be forecasted by parameters established in the laboratory? *Sci. Rep*. **6**, 37799; doi: 10.1038/srep37799 (2016).

**Publisher's note:** Springer Nature remains neutral with regard to jurisdictional claims in published maps and institutional affiliations.

## Figures and Tables

**Table 1:** Mean MTB values and standard deviations of the tested groups and statistical analyses within and between conditions (*in vivo* and *in vitro*).

Evaluated Period	Conditions			
	*In vivo*	*In vitro*		
	MTB	Simulated pulpal pressure	Aging	MTB
24 hours	23.21 ± 3.91^A, a^	No-SPP	Water ageing	24.96 + 3.00^A, a^
			Cyclic ageing	23.96 ± 1.77^A, a^
		SPP	Water ageing	17.65 ± 1.42^B, b^
			Cyclic ageing	19.49 ± 1.60^B, b^
6 months	14.17 ± 4.78^B, a^	No-SPP	Water ageing	16.68 ± 4.13^B, a^
			Cyclic ageing	18.17 ± 2.53^B, a^
		SPP	Water ageing	14.33 ± 4.69^B, a^
			Cyclic ageing	17.89 ± 3.98^B, a^

Different superscript capital letters denote differences within columns (within *in vivo* or *in vitro* conditions). Different superscript small letters denote differences within rows (comparison of *in vivo* with *in vitro* conditions in each evaluated period).

**Table 2:** Materials, manufacturer information, composition and batch number.

Material	Manufacturer	Composition	Batch number
Magic Acid Gel	Vigodent; Bonsucesso, Rio de Janeiro, Brazil	37% phosphoric acid	006/10
Adper Single Bond 2	3 M ESPE; St. Paul, MN, USA	BisGMA, HEMA, dimethacrylate, ethanol, water, photoinitiator, functional copolymer of methacrylate, polyacrylic acid and polyitaconic acid, silica nanoparticles	N289126BR
Grandio	Voco GmbH; Cuxhaven, Germany	BisGMA, TEGDMA (12%), inorganic particles (87%) and additive (1%)	1129360

Abbreviations. BisGMA: bisphenol A glycidyl dimethacrylate; HEMA: hydroxyethyl methacrylate.

TEGDMA: ethylene glycol dimethacrylate.
